# Socio-economic and demographic patterns of mental health complaints among the employed adults in Estonia

**DOI:** 10.1371/journal.pone.0258827

**Published:** 2021-10-25

**Authors:** Rainer Reile, Merike Sisask

**Affiliations:** 1 Department of Epidemiology and Biostatistics, National Institute for Health Development, Tallinn, Estonia; 2 Institute of Family Medicine and Public Health, University of Tartu, Tartu, Estonia; 3 Tallinn University, School of Governance, Law and Society, Tallinn, Estonia; 4 Estonian-Swedish Mental Health and Suicidology Institute (ERSI), Tallinn, Estonia; Neijiang Normal University, CHINA

## Abstract

**Background:**

Mental health problems follow a distinct socio-economic gradient and contribute to the health inequalities. The study aims to analyse the socio-economic and demographic factors of self-reported mental health complaints (stress, depressiveness, overtiredness, suicidal thoughts) among employed adult population in Estonia.

**Methods:**

Data on 4041 employed respondents (2064 men and 1977 women) aged 20–64 years from nationally representative health surveys from years 2016 and 2018 in Estonia were used for the study. Dependent variables included self-reported stress, depressiveness, overtiredness, and suicidal thoughts. Descriptive statistics and both log-binomial and Poisson regression analysis were used to describe the socio-economic and demographic variations in these mental health complaints.

**Results:**

More than half of the respondents had either stress, depressiveness, overtiredness or suicidal thoughts with 25% reporting two or more of mental health complaints. Lower personal income was associated with higher rates of all mental health complaints (stress, depressiveness, overtiredness, and suicidal thoughts) among employed adults in Estonia. Additionally, lower education was associated with higher prevalence of depressiveness and lower job skills predicted higher prevalence of suicidal thoughts. Higher prevalence ratios for depressiveness and overtiredness were found for women compared to men whereas Estonians had higher prevalence ratios for stress and suicidal thoughts compared to non-Estonians. All mental health complaints were more frequently reported at younger ages (compared to 50-64-year olds) and by not married or cohabiting respondents.

**Conclusion:**

High prevalence of mental health complaints and their socio-economic and demographic patterning refer to considerable inequalities in mental health among employed adults. Policy actions targeting especially younger adults and those with financial difficulties are needed to address these early manifestations of mental health problems.

## Introduction

Mental health problems in working aged population cause substantial loss in both health and economic terms [1] and are thus receiving increasing attention in the political agenda [1,2]. It is suggested that mental and addictive disorders affect more than 1 billion people globally causing about 7% of all global burden of disease as measured in disability-adjusted life-years (DALYs) and 19% of all years lived with disability (YLDs) [3]. Alternative estimates [4] point to figures nearly twice as high (13.0% of DALYs and 32% of YLDs), indicating that the overall disease burden of mental illness is likely to be underestimated.

Studies on the associations between mental health and employment status focus often on depression [5,6], anxiety [5,7,8], suicidality [9,10] and (di)stress [5,11]. Substantial amount of research has been dedicated to the associations between mental health and job insecurity, especially job loss and unemployment [6,7,12,13]. Although available evidence suggests that being employed has considerable mental health benefits over unemployment [14], there are also various underlying mechanisms for mental health problems among the employed. However, the employment and unemployment status couldn’t be viewed dichotomously, but rather in a continuum perspective with variations and heterogeneity in both statuses [5]. Besides being unemployed or underemployed, certain work-related life events can make people more vulnerable in terms of mental health, e.g. dismissal [15]. Even more, among those having secure employment position, several work-related factors like excessive workload, job strain, reward imbalance and low job satisfaction have been found to have adverse impact on mental health [16]. It is also known that mental health among working-age population can be influenced by uncertain times and increased job insecurity caused by societal level economic condition like economic recession or crisis [9,17].

In addition to overall socio-economic conditions, individual level socio-economic factors contribute to mental health outcomes as well [10,11]. There are several notions that mental health problems can be at least partially attributable to socio-economic inequalities due to educational background [5], occupational qualification [10,13], low income [8] and financial hardiness or poverty [12]. Among those, low income seems to be especially relevant in terms of predicting psychiatric comorbidity [8]. Education, however, is a significant predictor of employment status that partly explains the different mental health outcomes found by respondents’ educational level [5]. While the evidence on the relative effects of job skill level on the mental health of employed persons is inconclusive, occupations requiring lower qualifications are generally more at risk regarding mental health at work [18].

Demographic variables like age and sex may also predict poorer mental health status [13,17]. While these factors also partly underline the socio-economic inequalities in health, the associations between work-related conditions and mental health are often unaltered after controlling for these factors [19,20]. Their individual contribution is nonetheless significant. For example, Rudolph and Eaton [21] found that prior anxiety or depression increased risk of early labour force exit for women but not for men. In another study [22], higher vulnerability to depression during economic recession and unemployment was found among men and those between 35 and 49 years of age. Marital status is an important indicator of social and economic support and relationship context that has implications for mental health. The evidence suggests that divorced and single men have poorer health compared to their married counterparts (smaller differences are found for women) [23]. Additionally, ethnicity is a demographic characteristic that often translates into inequalities also in terms of mental health and working conditions. The higher risk of mental health problems among ethnic minorities compared to native population was demonstrated in a Dutch study [24] and more recently also in Estonian data [17] in the context of economic recession.

This study will focus on the potential socio-demographic patterning of mental health complaints among employed adult population. The aim of the study is to analyse the relative contribution of both individual socio-economic status and demographic background on the differences in perceived stress, depressiveness, overtiredness, and suicidal thoughts in Estonia.

## Methods

### Data collection and sample description

Data from Surveys of Health Behaviour among Estonian Adult Population conducted in 2016 and 2018 were used for the analysis. These cross-sectional postal and web-based surveys used nationally representative stratified random samples of 5000 individuals aged 16–64 years from Population Registry. Adjusted response rates were 56,5% (n = 2751) for 2016 and 51,4% (n = 2525) for 2018 surveys. Both surveys were approved by Tallinn Medical Research Ethics Committee. Detailed information about the surveys can be found elsewhere [25].

Current analysis uses data on 4041 (2064 men and 1977 women) respondents who were 20–64-year-old and were employed at the time of the surveys. The restriction was used to minimize the potential misclassification of socio-economic status among younger and non-employed respondents. Simple proportional population weights (based on 5-year age groups and gender distribution of Estonian population as of January 1^st^ for each study year) were used to compensate for the response bias and to assure that the data is representative for population aged 20–64 years in Estonia.

### Variables

The dependent variables were four items on self-reported mental health complaints: a) stress, b) depressiveness, c) overtiredness, and d) suicidal thoughts. Perceived stress was assessed with a question: “In the past 30 days, have you been stressed, under pressure?”. The response options were dichotomized into being: i) stressed (“yes, my life is almost unbearable” and “yes, more than people on the average”), and ii) not stressed (“yes, but no more than people on the average” and “no, not at all”). Depressiveness was assessed with a question: “In the past 30 days, have you been unhappy, depressed (suffering from depressiveness)?” Its response options were dichotomized into being: i) depressed (“yes, a lot more than before” and “yes, somewhat more than before”), and ii) not depressed (“yes, but no more than before” and “no, not at all”). Overtiredness was assessed with a question: “In the past 12 months, how often have you felt overtired?” with response options dichotomized into categories of: i) overtired (“almost all the time” or “quite often”) and ii) not tired (“seldom” or “never”). Suicidal thoughts were captured with a question: “Have you ever thought about suicide?” with response options dichotomized into categories of: i) suicidal thoughts (“yes, during the past 12 months”, “yes, earlier”, “yes, during the past 12 months and earlier”) and ii) no.

Respondents’ age, sex, ethnicity, and marital status were included as demographic variables. Age effects were analysed in groups of: i) 20–34, ii) 35–49, and iii) 50–64-year-olds (reference category). Data on self-reported ethnicity was grouped as: i) Estonians, and ii) non-Estonians (reference category). Marital status distinguished respondents who were: i) single (includes also divorced, separated, or widowed), or ii) married or cohabiting (reference category).

Socio-economic variables included education, income and job qualification. Educational level was measured by the highest level of education obtained and was categorized as i) primary education or less, ii) secondary or vocational, and iii) tertiary level education (reference category). Average net income per household member (measured initially on year-specific ordinal scale), was divided into quartiles and calculated separately for each study year. Job qualification was based on self-reported occupation measured with a scale following International Standard Classification of Occupations (ISCO) major groups. ISCO-08 classification (*International Standard Classification of Occupations 2008 (ISCO-08)*: *Structure*, *Group Definitions and Correspondence Tables* 2012) was used to assign these into four job skill groups (level I as the lowest and level IV as the highest). For managerial and armed forces occupations that cannot be assigned to job skill groups, educational level was used as suggested by ISCO-08.

### Data analysis

Characteristics of the study sample were summarized using descriptive statistics. Point prevalence with 95% confidence intervals (95% CI) was calculated as the proportion of cases reporting a mental health complaint divided by the total number of cases for different socio-economic and demographic strata. We used log-binomial regression to analyze the associations between reported mental health complaints (stress, depressiveness, overtiredness and suicidal thoughts) and independent demographic and socio-economic variables. Log-binomial model resembles logistic regression but use log link instead of logit link function. Several comparative studies [26,27] have demonstrated that odds ratios from logistic regression overestimate the association when the outcome is frequent. Log-binomial model with the outcome interpreted as prevalence ratios can be considered a suitable alternative in these cases. Additionally, a separate analysis was carried out to examine the socio-demographic patterning in co-existing mental health complaints. For this, log-linear Poisson regression was used with number of reported mental health complaints (range 0–4) being the dependent variable. In both analyses, Model 1 tested for the effects of all independent variables separately whereas all socio-economic and demographic variables were adjusted for each other in Model 2. For log-binomial models, the results are presented as exponentiated coefficients with 95% confidence intervals (CI) that can be interpreted as prevalence ratios (PR) with respective CIs. For Poisson models, the exponentiated coefficients with 95% confidence intervals (CI) are interpreted as rate ratios (RR) with respective CIs. In all cases, statistically significant (p-value <0.05) differences are marked in tables with asterisk. All statistical analyses were conducted using SPSS Statistics for Windows, version 25.0 (IBM Corp. 2017).

## Results

### Stress

Table 1 presents the prevalence estimates and associations with socio-economic and demographic variables for perceived stress. Statistically significant differences in stress prevalence were found for 20–34 olds, Estonians, and not married or cohabiting respondents.

**Table 1:** Stress and association with socio-economic and demographic variables, descriptive statistics and prevalence ratios with 95% CIs.

		Descriptive statistics		Model 1^a^	Model 2^b^
		n	Prevalence, % (95% CI)	PR (95% CI)	PR (95% CI)
*Socio-economic variables*					
Education	Primary or less	401	21.7 (17.9–25.9)	1.04 (0.84–1.29)	1.11 (0.87–1.42)
	Secondary or vocational	2009	17.8 (16.2–19.5)	0.86 (0.75–0.98)*	0.93 (0.79–1.10)
	Tertiary	1577	20.8 (18.8–22.8)	1	1
Income	1st quartile (lowest)	773	22.3 (19.4–25.3)	1.07 (0.89–1.29)	1.35 (1.11–1.66)*
	2nd quartile	1272	17.5 (15.5–19.7)	0.83 (0.69–0.98)*	0.99 (0.82–1.20)
	3rd quartile	1024	18.8 (16.4–21.2)	0.87 (0.72–1.05)	0.96 (0.79–1.16)
	4th quartile (highest)	831	20.8 (18.2–23.7)	1	1
Job skill	Skill lvl I (lowest)	242	17.6 (13.4–23.0)	0.83 (0.62–1.11)	0.82 (0.59–1.13)
	Skill lvl II	1855	18.8 (17.1–20.6)	0.90 (0.77–1.04)	0.90 (0.75–1.09)
	Skill lvl III	714	18.3 (15.6–21.3)	0.85 (0.70–1.03)	0.87 (0.71–1.07)
	Skill lvl IV (highest)	995	21.6 (19.1–24.2)	1	1
*Demographic variables*					
Age	20–34	1357	26.0 (23.8–28.5)*	1.90 (1.62–2.24)*	1.83 (1.55–2.17)*
	35–49	1454	18.0 (16.0–20.0)*	1.31 (1.10–1.56)*	1.23 (1.03–1.47)*
	50–64	1180	13.4 (11.6–15.5)	1	1
Sex	Female	1958	19.9 (18.2–21.7)	1.05 (0.93–1.19)	1.11 (0.97–1.27)
	Male	2034	18.9 (17.2–20.6)	1	1
Ethnicity	Estonian	2980	21.3 (19.8–22.8)*	1.51 (1.28–1.77)*	1.53 (1.28–1.82)*
	Other	1011	13.8 (11.7–16.0)	1	1
Marital status	Single/divorced/separa-ted/widow	1144	23.6 (21.2–26.1)*	1.34 (1.18–1.52)*	1.31 (1.14–1.49)*
	Married/cohabiting	2831	17.6 (16.3–19.1)	1	1

^a^ Univariate model.

^b^ Mutually adjusted model.

* Statistically significant difference (p<0.05) compared to the reference category.

Univariate regression analysis (Model 1) found statistically significant (p<0.05) differences for education, income, age, ethnicity, and marital status. After mutual adjustment for other variables (Model 2), among socio-economic variables only the difference between the lowest and the highest income remained statistically significant, those with the lowest income had higher prevalence of stress compared to respondents with highest income (PR 1.35; 95% CI 1.11–1.66). In addition to lower income, younger age (for 20-34-year-olds PR 1.83; 95% CI 1.55–2.17; and for 35-49-year olds PR 1.23; CI 1.03–1.47), Estonian ethnicity (PR 1.53; 95% CI 1.28–1.82) and being single (PR1.31; 95% CI 1.14–1.49) predicted higher prevalence of stress in Model 2.

### Depressiveness

Prevalence of depressiveness (Table 2) was higher among respondents with lowest education and income, for 20–34 olds, females and those not married or cohabiting.

**Table 2:** Perceived depressiveness and association with socio-economic and demographic variables, descriptive statistics and prevalence ratios with 95% CIs.

		Descriptive statistics		Model 1^a^	Model 2^b^
		n	Prevalence, % (95% CI)	PR (95% CI)	PR (95% CI)
*Socio-economic variables*					
Education	Primary or less	402	21.1 (17.4–25.3)*	1.38 (1.10–1.73)*	1.33 (1.02–1.73)*
	Secondary or vocational	2011	17.1 (15.5–18.7)	1.11 (0.96–1.29)	1.11 (0.92–1.34)
	Tertiary	1574	14.7 (13.1–16.6)	1	1
Income	1st quartile (lowest)	774	20.9 (18.2–23.9)*	1.38 (1.12–1.70)*	1.42 (1.13–1.80)*
	2nd quartile	1268	16.9 (14.9–19.0)	1.10 (0.90–1.39)	1.17 (0.94–1.45)
	3rd quartile	1025	14.6 (12.5–16.8)	0.96 (0.77–1.19)	0.99 (0.79–1.24)
	4th quartile (highest)	832	15.2 (12.9–17.8)	1	1
Job skill	Skill lvl I (lowest)	241	17.4 (13.0–22.6)	1.18 (0.87–1.62)	0.97 (0.69–1.38)
	Skill lvl II	1858	17.9 (16.2–19.7)	1.26 (1.05–1.50)*	1.09 (0.87–1.36)
	Skill lvl III	712	15.2 (12.7–17.9)	1.02 (0.81–1.29)	0.96 (0.76–1.22)
	Skill lvl IV (highest)	994	14.1 (12.0–16.4)	1	1
*Demographic variables*					
Age	20–34	1362	22.7 (20.6–25.0)*	1.78 (1.50–2.12)*	1.87 (1.56–2.23)*
	35–49	1450	14.3 (12.5–16.1)	1.16 (0.96–1.40)	1.16 (0.95–1.40)
	50–64	1180	12.2 (10.4–14.2)	1	1
Sex	Female	1954	18.5 (16.9–20.3)*	1.27 (1.10–1.47)*	1.34 (1.18–1.59)*
	Male	2038	14.7 (13.2–16.3)	1	1
Ethnicity	Estonian	2980	17.0 (15.7–18.4)	1.09 (0.93–1.28)	1.10 (0.93–1.29)
	Other	1012	15.3 (13.2–17.6)	1	1
Marital status	Single/divorced/separated/widow	1145	22.0 (19.7–24.5)*	1.54 (1.34–1.77)*	1.40 (1.22–1.62)*
	Married/cohabiting	2830	14.3 (13.1–15.7)	1	1

^a^ Univariate model.

^b^ Mutually adjusted model.

* Statistically significant difference (p<0.05) compared to the reference category.

In univariate model, statistically significant (p<0.05) differences were found for age, sex, marital status, education, income, and job skills. Differences in depressiveness by education and income were found also in mutually adjusted regression analysis (Model 2), where those with primary education or less (PR 1.33; 95% CI 1.02–1.73) compared to those with tertiary education and the lowest income (PR 1.42; 95% CI 1.13–1.80) compared to those with the highest income having significantly higher depressiveness rates. For demographic variables, higher prevalence rates of depressiveness were found among 20-34-year-old (PR 1.87; 95% CI 1.56–2.23), female (PR 1.34; 95% CI 1.18–1.59) and single (PR 1.40; 95% CI 1.22–1.62) respondents.

### Overtiredness

Overtiredness (Table 3) was the most frequently reported mental health complaint with the prevalence exceeding 40% in almost all demographic and socio-economic groups. However, statistically significant differences in prevalence of overtiredness were found for lowest income quartile and females. Univariate regression analysis (Model 1) found that statistically significant differences in prevalence of overtiredness were found for education, income, age, sex, and marital status. In mutually adjusted model (Model 2) the income remained statistically significant (for 1^st^ quartile PR 1.23; 95% CI 1.09–1.38; and for 2^nd^ quartile PR 1.11; 95% CI 1.00–1.24). For demographic variables, higher rates of overtiredness were reported by younger respondents (for 20-34-year-olds PR 1.22; 95% CI 1.11–1.33; for 35-49-year-olds PR 1.11; 95% CI 1.02–1.22) and single respondents (PR 1.08; 95% CI 1.00–1.16). Females had higher rates of overtiredness (PR 1.30; 95% CI 1.21–1.41).

**Table 3:** Overtiredness and association with socio-economic and demographic variables, descriptive statistics and prevalence ratios with 95% CIs.

		Descriptive statistics		Model 1^a^	Model 2^b^
		n	Prevalence, % (95% CI)	PR (95% CI)	PR (95% CI)
*Socio-economic variables*					
Education	Primary or less	401	48.3 (43.3–53.0)	1.12 (1.00–1.26)*	1.12 (0.98–1.28)
	Secondary or vocational	2018	44.5 (42.4–46.7)	1.04 (0.97–1.12)	1.04 (0.95–1.14)
	Tertiary	1573	42.6 (40.2–45.0)	1	1
Income	1st quartile (lowest)	774	50.9 (47.4–54.4)*	1.28 (1.15–1.42)*	1.23 (1.09–1.38)*
	2nd quartile	1277	44.8 (42.2–47.6)	1.13 (1.02–1.25)*	1.11 (1.00–1.24)*
	3rd quartile	1021	41.9 (38.9–45.0)	1.06 (0.95–1.18)	1.05 (0.94–1.17)
	4th quartile (highest)	831	39.5 (36.2–42.8)	1	1
Job skill	Skill lvl I (lowest)	241	46.0 (39.8–52.4)	1.13 (0.98–1.31)	1.08 (0.92–1.27)
	Skill lvl II	1860	45.5 (43.3–47.8)	1.08 (0.99–1.17)	1.00 (0.90–1.11)
	Skill lvl III	713	42.2 (38.6–45.9)	0.98 (0.88–1.10)	0.95 (0.85–1.07)
	Skill lvl IV (highest)	995	42.0 (39.0–45.1)	1	1
*Demographic variables*					
Age	20–34	1359	46.5 (43.9–49.2)	1.14 (1.05–1.24)*	1.22 (1.11–1.33)*
	35–49	1453	44.3 (41.8–46.9)	1.09 (1.00–1.19)	1.11 (1.02–1.22)*
	50–64	1184	41.1 (38.4–44.0)	1	1
Sex	Female	1964	50.0 (47.8–52.3)*	1.28 (1.20–1.39)*	1.30 (1.21–1.41)*
	Male	2032	38.4 (36.3–40.5)	1	1
Ethnicity	Estonian	2983	43.8 (42.0–45.6)	0.96 (0.89–1.04)	0.98 (0.90–1.06)
	Other	1013	45.0 (42.0–48.1)	1	1
Marital status	Single/divorced/separated/widow	1148	46.9 (44.1–49.8)	1.09 (1.02–1.18)*	1.08 (1.00–1.16)*
	Married/cohabiting	2832	43.0 (41.2–44.8)	1	1

^a^ Univariate model.

^b^ Mutually adjusted model.

* Statistically significant difference (p<0.05) compared to the reference category.

### Suicidal thoughts

The prevalence of suicidal thoughts (Table 4) was higher among those with the lowest income and job skill level, younger respondents, Estonians, and female respondents. Statistically significant (p<0.05) differences (Model 1) were found for income, job skill, age, ethnicity, and marital status. The differences in socio-economic variables persisted in mutually adjusted regression model (Model 2) with respondents from lower income quartiles (for 1^st^ quartile PR 1.57; 95% CI 1.21–2.06; for 2^nd^ quartile PR 1.39; 95% CI 1.09–1.78) and with lower job skill (for skill level I PR 1.60; 95% CI 1.12–2.30; for skill level II PR 1.30; 95% CI 1.01–1.65) having higher rates of suicidal thoughts. The demographic differences in suicidal thoughts are also noteworthy: younger age groups (for 20-34-year-olds PR 1.91; 95% CI 1.55–2.36; for 35-49-year-olds PR 1.44; 95% CI 1.16–1.79), Estonians (PR 1.80; 95% CI 1.44–2.25) and single respondents (PR 1.52; 95% CI 1.29–1.78) having higher prevalence of suicidal thoughts.

**Table 4:** Suicidal thoughts and association with socio-economic and demographic variables, descriptive statistics and prevalence ratios with 95% CIs.

		Descriptive statistics		Model 1^a^	Model 2^b^
		n	Prevalence, % (95% CI)	PR (95% CI)	PR (95% CI)
*Socio-economic variables*					
Education	Primary or less	403	16.3 (13.0–20.2)	1.20 (0.93–1.55)	0.93 (0.68–1.26)
	Secondary or vocational	2013	13.8 (12.4–15.4)	1.03 (0.88–1.22)	0.88 (0.72–1.09)
	Tertiary	1575	13.3 (11.7–15.1)	1	1
Income	1st quartile (lowest)	777	16.9 (14.4–19.6)*	1.44 (1.13–1.84)*	1.57 (1.21–2.06)*
	2nd quartile	1273	15.2 (13.3–17.2)	1.28 (1.02–1.60)*	1.39 (1.09–1.78)*
	3rd quartile	1024	12.2 (10.3–14.3)	1.04 (0.81–1.33)	1.09 (0.84–1.40)
	4th quartile (highest)	831	11.5 (9.40–13.7)	1	1
Job skill	Skill lvl I (lowest)	240	18.8 (14.2–24.0)*	1.62 (1.18–2.21)*	1.60 (1.12–2.30)*
	Skill lvl II	1859	14.4 (12.9–16.1)	1.31 (1.07–1.60)*	1.30 (1.01–1.65)*
	Skill lvl III	714	12.9 (10.6–15.5)	1.11 (0.86–1.44)	1.10 (0.85–1.44)
	Skill lvl IV (highest)	995	11.5 (9.6–13.5)	1	1
*Demographic variables*					
Age	20–34	1362	18.1 (16.2–20.2)*	1.94 (1.58–2.37)*	1.91 (1.55–2.36)*
	35–49	1454	13.6 (11.9–15.4)*	1.45 (1.17–1.79)*	1.44 (1.16–1.79)*
	50–64	1180	9.4 (7.8–11.1)	1	1
Sex	Female	1958	14.6 (13.0–16.2)	1.09 (0.93–1.27)	1.14 (0.96–1.33)
	Male	2038	13.2 (11.8–14.8)	1	1
Ethnicity	Estonian	2982	15.4 (14.2–16.8)*	1.66 (1.39–1.88)*	1.80 (1.44–2.25)*
	Other	1014	9.3 (7.7–11.3)	1	1
Marital status	Single/divorced/separated/widow	1148	19.2 (17.0–21.5)*	1.62 (1.29–1.78)*	1.52 (1.29–1.78)*
	Married/cohabiting	2832	11.8 (10.6–13.0)	1	1

^a^ Univariate model.

^b^ Mutually adjusted model.

* Statistically significant difference (p<0.05) compared to the reference category.

### Co-existing mental health complaints

Mental health complaints–either stress, depressiveness, overtiredness or suicidal thoughts–were reported by 53% of 20–64 year old employed adults in Estonia with nearly half of them having two or more complaints. Distribution of reported co-existing mental health complaints by socio-economic and demographic variables is presented in Table 5.

**Table 5:** Distribution of reported co-existing mental health complaints by socio-economic and demographic variables.

		n	No of complaints reported, %				
			0	1	2	3	4
*Socio-economic variables*							
Education	Primary or less	398	42.6	27.8	15.0	8.3	6.2*
	Secondary or vocational	1990	46.9	28.8	11.9	9.0	3.3
	Tertiary ^a^	1568	48.2	26.3	14.4	7.9	3.1
Income	1st quartile (lowest)	767	40.6*	29.2*	14.0	10.6	5.7*
	2nd quartile	1260	46.1	29.8*	12.0	8.3	3.8
	3rd quartile	1014	48.5	28.1	13.7	6.8	2.9
	4th quartile (highest) ^a^	829	51.8	22.7	14.3	9.3	1.9
Job skill	Skill lvl I (lowest)	238	43.2	30.0	14.9	7.8	4.1
	Skill lvl II	1839	45.9	28.5	12.8	8.8	4.0
	Skill lvl III	711	49.4	26.6	12.2	9.1	2.7
	Skill lvl IV (highest) ^a^	989	49.3	25.6	14.7	7.7	2.7
*Demographic variables*							
Age	20–34	1352	41.5*	26.1	15.7*	10.8	5.9*
	35–49	1444	47.1*	29.1	13.0	8.2	2.6
	50–64 ^a^	1164	53.3	28.0	10.5	6.2	2.0
Sex	Female	1941	42.3*	29.9*	14.4	9.6	3.8
	Male ^a^	2019	51.6	25.6	12.1	7.5	3.2
Ethnicity	Estonian	2959	46.4	26.7	13.9	8.9	4.1*
	Other ^a^	1001	48.7	30.8	11.1	7.4	1.9
Marital status	Single/divorced/separated/widow	1138	41.7*	27.8	13.7	11.1*	5.8*
	Married/cohabiting ^a^	2807	49.2	27.7	13.0	7.4	2.7

^a^ reference category for Chi-square tests within category of reported mental health complaints.

Table 6 presents the results from Poisson regression for the associations between number of reported mental health complaints and socio-economic and demographic variables. In Model 1, statistically significant (p<0.05) differences were found for all demographic variables and for differences between lowest and highest education and income groups and 2^nd^ vs 4^th^ job skill level groups. Except for income, the differences in socio-economic variables were attenuated to non-significant level in mutually adjusted model (Model 2). For example, those with lowest income had 1.35 times (95% CI 1.19–1.53, p<0.001) more mental health complaints compared to highest income group given the other variables are held constant. However, the effects of demographic variables were substantially stronger in predicting the number of mental health complaints. For example, respondents aged 20–34 years have 53% higher risk for reporting higher number of mental health complaints compared to 50–64 year old. Statistically significant differences were also found for age group 35–49 (RR 1.18; 95% CI 1.07–1.31), females (RR 1.24; 95% CI 1.15–1.34), Estonians (RR 1.17; 95% CI 1.08–1.29) and not married or cohabiting respondents (RR 1.24; 95% CI 1.14–1.34).

**Table 6:** Association of co-existing mental health complaints by socio-economic and demographic variables, rate ratios with 95% CIs.

		Model 1^a^		Model 2^b^
			RR (95% CI)	RR (95% CI)
*Socio-economic variables*				
Education	Primary or less	360	1.21 (1.06–1.37)*	1.15 (0.99–1.32)
	Secondary or vocational	1890	1.03 (0.95–1.11)	1.01 (0.91–1.11)
	Tertiary	1564	1	1
Income	1st quartile (lowest)	735	1.30 (1.16–1.46)*	1.35 (1.19–1.53)*
	2nd quartile	1274	1.09 (0.97–1.21)	1.13 (1.01–1.27)*
	3rd quartile	1002	1.00 (0.89–1.13)	1.02 (0.91–1.15)
	4th quartile (highest)	803	1	1
Job skill	Skill lvl I (lowest)	242	1.13 (0.97–1.33)	1.05 (0.88–1.25)
	Skill lvl II	1845	1.11 (1.01–1.22)*	1.03 (0.92–1.15)
	Skill lvl III	726	1.00 (0.89–1.12)	0.95 (0.85–1.08)
	Skill lvl IV (highest)	1001	1	1
*Demographic variables*				
Age	20–34	1175	1.49 (1.36–1.64)*	1.53 (1.39–1.68)*
	35–49	1382	1.18 (1.08–1.30)*	1.18 (1.07–1.31)*
	50–64	1257	1	1
Sex	Female	2141	1.21 (1.12–1.30)*	1.24 (1.15–1.34)*
	Male	1673	1	1
Ethnicity	Estonian	2853	1.15 (1.06–1.26)*	1.17 (1.08–1.29)*
	Other	961	1	1
Marital status	Single/divorced/separated/widow	1068	1.29 (1.19–1.40)*	1.24 (1.14–1.34)*
	Married/cohabiting	2746	1	1

^a^ Univariate model.

^b^ Mutually adjusted model.

* Statistically significant difference (p<0.05) compared to the reference category.

## Discussion

This study examined the socio-demographic patterning of mental health complaints among employed adult population in Estonia. The mental health complaints follow a distinct socio-economic and demographic pattern. Low personal income is associated with all mental health complaints considered–higher rates of stress, depressiveness, overtiredness, and suicidal thoughts. In addition to income, low education is linked with higher prevalence of depressiveness and low job qualification predicts higher prevalence of suicidal thoughts among employed adults in Estonia. Moreover, all mental health complaints are more frequently reported at younger ages and by not married or cohabiting respondents. Depressiveness and overtiredness are patterned by gender (women having higher probability) whereas stress and suicidal thoughts are more prevalent among Estonians. We also analysed the co-existence of the mental health complaints and found that more than half of the respondents reported at least one complaint and in turn nearly half of those reporting any complaints had two or more of them. However, the overall pattern of associations with socio-economic and demographic variables was quite similar to mental health complaints analysed one by one: higher risk for multiple mental health complaints was associated with lower income, younger age, female sex, being Estonian and not being married or cohabiting.

Before discussing these findings in detail, some aspects regarding the data and methods used should be considered. First, the surveys relied on self-reported mental health indicators. As these items do not allow establishing clinical diagnosis of stress-related disorders or depression, the reported prevalence reflects the subjective presence of these complaints. Second, a potential limitation is the lack of variables to control for work characteristics as such items were not included in the survey questionnaires. Previous studies among employed (e.g. [19,28]) suggest that work characteristics such as level of responsibility, overemployment and low reward contribute to poor mental health. However, it can be assumed that part of this variation is mediated via socio-economic variables in our study (especially job skill level and income reflected in it). However, despite the inclusion of different demographic and socio-economic variables, it is unlikely that the set of variables accounts for total variance in the dependent variable. Thus potential residual bias should be considered when interpreting the results. Third, given the study’s focus on socio-economic determinants of mental health outcomes, restrictions based on age and employment status had to be made to the study sample. To ensure sufficient statistical power, data from two consecutive surveys in 2016 and 2018 were pooled. The respective period changes in the outcome measures are therefore not covered in current analysis but can be found elsewhere [25]. Fourth, the relatively modest response rates of the surveys required population weighting to reduce the potential non-response bias and to assure the representativeness of the data. Finally, the cross-sectional study design does not allow to establish causality between socio-economic and demographic indicators and mental health complaints. Despite these considerations, the standardised methodology and survey data are the major strengths of this study as it provides insight into the mental health characteristics of a nationally representative sample of working-aged, employed population. To the best of our knowledge this is the first study on the socio-demographic inequalities in mental health among the employed from Eastern Europe providing thus new insights to the problem in this region.

Earlier studies [12,29] have mentioned financial hardship, sense of mastery, and social support as key variables that explain the association between employment status and poor mental health. Similar concepts, represented by low income, younger age and being not married/cohabiting, were also highly significant in our analysis. The financial hardship can be interpreted as having limited monetary resources for daily needs or having low income as in our case. Out of the socio-economic variables covered, low personal income was the strongest predictor of mental health among employed persons as it correlated with all mental health variables in our study. The finding on the relative importance of income on mental health is in accordance with several previous studies based on working-age samples [8,17,30]. For example, in a study by Pulkki-Råback et al [8], low income was associated with increased risk for depressive disorder (OR 1.73) and anxiety disorder (OR 1.56). As income-effects on mental health complaints in our study were statistically significant only for the lowest quartile (for overtiredness and suicidal thoughts also for the 2^nd^ quartile), the results suggest that absolute rather than relative deprivation is detrimental for mental health outcomes in this context.

Other socio-economic variables had relatively modest effects on mental health complaints. Low education was associated with higher prevalence of depressiveness and lower categories of job skill levels with higher prevalence of suicidal thoughts in the final model. However, this does not necessarily mean that they were less important characteristics than personal income, as education and high qualification are prerequisites for having high income. Educational achievement has been associated with reduced risk of co-occurring mental health and substance use problems that relate to economic instability in young adulthood [31]. Educational achievement also relates to sense of mastery as demonstrated in another study [32] where sense of mastery accounted for about half of the variation between level of education and psychological distress.

Demographic characteristics, especially age and marital/cohabitation status were highly relevant in terms of mental health. Younger age and being single was associated with elevated risk of perceived stress, depressiveness, overtiredness and also for suicidal thoughts. In the age group of 20-34-year olds, every fourth reported elevated stress and almost every fifth has had suicidal thoughts. Compared to 50-64-year olds, the difference was nearly twofold. One likely explanation for higher prevalence (and susceptibility) of mental health complaints relates to experience of major life transitions that are embedded in changing social contexts when completing school, leaving home and entering the workforce [33]. These transitions are affected by prior mental health and shape mental health at older ages. As the survey design did not allow to study the timing and duration of the employment status, further research along these lines is warranted. However, previously mentioned concepts of financial hardship, sense of mastery, and social support are also relevant in explaining these demographic variances. In this context, differences in mental health complaints by marital status stand out: those who were married/cohabiting had significantly lower prevalence of all mental health complaints considered in our study. It is known that having close relationships increases psychological, social, and economic resources [34] and helps to alleviate the negative effects of employment-related stressors. In the context of employment status, low employment stability has been associated with poorer mental health status among single men and women [35].

Compared to age and marital status, the effects of sex and ethnicity of the respondent on the mental health outcomes were less pronounced. Depressiveness and overtiredness are patterned by gender, whereas stress and suicidal thoughts are more prevalent among Estonians. While higher prevalence of mental health complaints among women is previously known [36], the differences in work-related stressors are one potential explanation. In a recent study on recession-related mental health effects in Estonia [17], the higher period-increase in prevalence of depression for men was explained by employment changes in the construction and manufacturing sectors where men havetraditionally had a larger share of jobs. However, in case of employed sample, socio-economic factors as well as work-characteristics and amount of household work could explain the higher prevalence of stress and depression among women [37].

Although ethnic minorities have been previously associated with higher risk of mental health problems in some European countries [24], the opposite was found in our study. The current ethnic composition in Estonia was formed during the Soviet period and even today the subsequent generations have quite strong ethnic identity and are not well integrated to the society [38]. A previous study has identified an interesting pattern in suicidal behaviour among Russians in Estonia [39]. During the Soviet period the suicide rate among the Russian minority in Estonia was lower than the rate in native Estonians, but it became significantly higher in independent Estonia in 1990s, when Russians changed from a privileged to a non-privileged minority. Thus, in terms of mental health, the ethnic minority in Estonia may not comply with the patterns known from other European countries.

The data of the current study was collected during the period before COVID-19. However, several recently published studies have shown that the effect of COVID-19 lockdown can be devastating to mental health, especially in working life contexts [40,41]. Forthcoming study waves within the Surveys of Health Behaviour among Estonian Adult Population could potentially provide material to for analyzing employed adults’ mental health complaints before and after the COVID-19.

## Conclusion

These findings outline considerable socio-economic and demographic variations in mental health complaints (stress, depressiveness, overtiredness, suicidal thoughts) among employed adult population. The study demonstrates widespread and relatively steep socio-economic and demographic inequalities in mental health among the employed. While demographic factors such as younger age, being female, Estonian and not married or cohabiting constitute elevated risk for having poorer mental health, having low income is a significant predictor for mental health complaints as well. The importance of self-reported mental health complaints, the pre-clinical manifestations of possible mental disorders should not be underestimated. Early recognition and intervention at workplaces could reduce the onset of mental disorders and thus reduce the overall disease burden and economic costs at the population level. Initiatives providing short-term social and economic support for those experiencing financial difficulties, especially at younger age groups, are warranted to reduce current inequalities in mental health among working age population, especially under the impact of COVID-19 situation.
